# Improved hospital-level risk adjustment for surveillance of healthcare-associated bloodstream infections: a retrospective cohort study

**DOI:** 10.1186/1471-2334-9-145

**Published:** 2009-09-01

**Authors:** ENC Tong, ACA Clements, MA Haynes, MA Jones, AP Morton, M Whitby

**Affiliations:** 1 Centre for Healthcare Related Infection Surveillance and Prevention, Royal Brisbane & Women's Hospital, Brisbane, Australia; 2 University of Queensland, School of Population Health, Brisbane, Australia; 3 Australian Centre for International and Tropical Health, Queensland Institute of Medical Research, Brisbane, Australia; 4 University of Queensland, The Institute for Social Science Research, Brisbane, Australia; 5 Infection Management Services, Princess Alexandra Hospital, Brisbane, Australia

## Abstract

**Background:**

To allow direct comparison of bloodstream infection (BSI) rates between hospitals for performance measurement, observed rates need to be risk adjusted according to the types of patients cared for by the hospital. However, attribute data on all individual patients are often unavailable and hospital-level risk adjustment needs to be done using indirect indicator variables of patient case mix, such as hospital level. We aimed to identify medical services associated with high or low BSI rates, and to evaluate the services provided by the hospital as indicators that can be used for more objective hospital-level risk adjustment.

**Methods:**

From February 2001-December 2007, 1719 monthly BSI counts were available from 18 hospitals in Queensland, Australia. BSI outcomes were stratified into four groups: overall BSI (OBSI), *Staphylococcus aureus *BSI (STAPH), intravascular device-related *S. aureus *BSI (IVD-STAPH) and methicillin-resistant *S. aureus *BSI (MRSA). Twelve services were considered as candidate risk-adjustment variables. For OBSI, STAPH and IVD-STAPH, we developed generalized estimating equation Poisson regression models that accounted for autocorrelation in longitudinal counts. Due to a lack of autocorrelation, a standard logistic regression model was specified for MRSA.

**Results:**

Four risk services were identified for OBSI: AIDS (IRR 2.14, 95% CI 1.20 to 3.82), infectious diseases (IRR 2.72, 95% CI 1.97 to 3.76), oncology (IRR 1.60, 95% CI 1.29 to 1.98) and bone marrow transplants (IRR 1.52, 95% CI 1.14 to 2.03). Four protective services were also found. A similar but smaller group of risk and protective services were found for the other outcomes. Acceptable agreement between observed and fitted values was found for the OBSI and STAPH models but not for the IVD-STAPH and MRSA models. However, the IVD-STAPH and MRSA models successfully discriminated between hospitals with higher and lower BSI rates.

**Conclusion:**

The high model goodness-of-fit and the higher frequency of OBSI and STAPH outcomes indicated that hospital-specific risk adjustment based on medical services provided would be useful for these outcomes in Queensland. The low frequency of IVD-STAPH and MRSA outcomes indicated that development of a hospital-level risk score was a more valid method of risk adjustment for these outcomes.

## Background

Healthcare-acquired infection (HAI) is a major contributor to patient morbidity and mortality [1], particularly bloodstream infections (BSI), which are expensive and difficult to treat [2]. Queensland Health has initiated a quality improvement programme, the Centre for Healthcare Related Infection Surveillance and Prevention (CHRISP), which undertakes standardized surveillance of HAI in public hospitals in Queensland.

To allow direct comparison of rates of HAI between hospitals, observed rates need to be risk adjusted according to the types of patients cared for by the hospital [3]. Without risk adjustment, hospitals might be penalized for high infection rates that arise due to the type of patients cared for rather than quality of patient care [4]. For surgical site infections, this involves risk adjusting individual patient outcomes according to measures of health status and surgical complexity [5]. However, for BSI, no individual data are collected by CHRISP on the general patient population, meaning that risk adjustment for hospital BSI rates has to be done indirectly, based on attributes of the hospital.

At present, expected BSI rates are crudely calculated for three hospital strata (called "levels") which roughly correspond to the size of, and types of services provided by, the facility. Level I hospitals are tertiary teaching hospitals, level II hospitals are large general hospitals and level III hospitals are smaller general hospitals. Level I hospitals tend to have higher rates of BSI than levels II and III hospitals, and crude risk adjustment based on hospital level allows for some of the between-hospital variation associated with patient case mix to be accounted for. However, CHRISP is seeking a more objective approach to risk adjustment based on hospital attributes (i.e. services provided) that are directly associated with BSI risk. The aim of the present study was to identify hospital services associated with high or low rates of BSI and to evaluate the services as indicators that can be used for improved hospital-level risk adjustment.

## Methods

CHRISP was initiated in 2000 with joint funding from the Australian Government Department of Health and Ageing, and Queensland Health, the Queensland government public health service. Surveillance methods are described in detail elsewhere [6], but here we provide a brief description of CHRISP surveillance of BSI. BSI data collection commenced in February 2001 on a voluntary basis and involved the 10 largest public hospitals in Queensland. In May 2002, an additional 11 smaller general hospitals were included. HAI (including BSI) data were collected by infection control practitioners in each participating hospital using hand-held computing devices. Standard BSI definitions based on the United States National Nosocomial Infection Surveillance (NNIS) system definitions were used in all hospitals [7]. Patient de-identified data were transferred to an electronic surveillance software package, Electronic Infection Control Assessment Technology version 4.2. (eICAT, CHRISP, Brisbane, Australia) from which the data for this study were extracted. Ultimately, data were available from 5 level I, 10 level II and 6 level III public hospital.

### Statistical analysis

Four types of BSI were investigated: Overall BSI (OBSI); BSI caused by *Staphylococcus aureus *(STAPH); Intravascular-device-related *S. aureus *BSI (IVD-STAPH) and BSI caused by methicillin-resistant *S. aureus *(MRSA), with the latter two forming overlapping subsets of STAPH and STAPH forming a subset of OBSI. As the frequency of MRSA monthly counts (number of infections per month) was low, with only 6.3 percent of all MRSA events being multiple events in the same month, this outcome was dichotomized to presence or absence of infections in each hospital and month. All BSI infection data were collected at an aggregated hospital level every month.

Split-sample validation was employed in the analysis. The training dataset consisted of a retrospective cohort of hospital-level monthly counts, comprising almost six years (71 months) of longitudinal data, collected from February 2001 to December 2006. The validation dataset comprised one year of longitudinal data, collected from January to December 2007. Three level II hospitals with multiple periods of missing longitudinal data were removed prior to analysis. The remaining 18 hospitals also had 11.3 percent missing outcome data because not all of these hospitals had joined CHRISP and began contributing data at the same time. However, these hospitals did not have missing data from the period that they started contributing. The training dataset had a total of 1122 observations.

Generalized estimating equation (GEE) Poisson regression models, typically used to compute population-averaged parameter estimates, were developed to identify risk and protective services for the OBSI, STAPH and IVD-STAPH outcome. The total number of patient days per month was used as an exposure variable in the models to capture the activity level of the hospital in a particular month. We used the quasilikelihood under the independence model information criterion (QIC), which is analogous to the Akaike information criterion (AIC) [8] for likelihood-based models, to select a parsimonious model with the best fitting temporal autocorrelation structure. As for AIC, a lower QIC indicates a better trade-off between model complexity and fit [9-11]. For the dichotomous MRSA outcome, an independence logistic regression model was developed to identify risk factor services.

Saturated models with the following 12 candidate medical services were fitted: acute renal dialysis, acquired immune deficiency syndrome (AIDS), alcohol/drugs, cardiac surgery, diabetes, hospice care, infectious diseases, intensive care, plastic surgery, obstetrics and maternity, oncology and bone marrow transplants. Five candidate medical services were excluded due to collinearity: acute spinal injury, burns, neurosurgery, obstetrics and intensive care. Collinearity arose for a medical service when there was minimal variation in that service across hospitals. For example, the intensive care service was collinear because it was offered by most hospitals and had a similar distribution across hospitals to the infectious diseases service. The general surgery service was also excluded because it was provided by all hospitals.

Parsimonious models were sought by dropping non-significant medical services using an *α*-level of 0.05. Parameter estimates for the GEE Poisson regression models were expressed in terms of incidence rate ratios (IRR) and 95% confidence intervals. Parameter estimates for the logistic regression model were expressed in terms of odds ratios (OR) and 95% confidence intervals.

### Goodness of fit analysis

For the count outcomes, the concordance correlation was computed as a measure of agreement between the observed and fitted values [12-14]. High levels of agreement implied that the model's fitted values closely matched the observed values. The Harrell's *c*-index was also derived, as a measure of discrimination between hospitals with higher or lower infection rates. For dichotomous outcomes, the *c*-index is equivalent to the area under the ROC curve (AUC).

For the dichotomous MRSA outcome, a Hosmer-Lemeshow test with 10 groups was performed to assess the logistic regression model. A *p*-value greater than .05 indicated no statistical evidence of a poorly fitting model. Receiver operating characteristics (ROC) analysis was conducted and the AUC was computed. The AUC measured discrimination, which is the ability of the model to correctly predict the months with and without infections. An AUC of 0.5 represented a model that predicts no better than random guessing and an AUC of 1 represented a model that predicts perfectly.

### Level re-classification based on risk scoring

For count outcomes where the models had a low concordance correlation, use of the regression model coefficients to calculate expected rates for direct hospital-level risk adjustment was not indicated. For these outcomes, and for the MRSA outcome, which occurred with a low frequency, an alternative risk-scoring approach [15] was explored. In this approach, a risk score that reflected the high and low risk services provided by a particular hospital was calculated by totaling the regression coefficients from the applicable medical services provided by that hospital. So, a hospital with an infectious diseases and cardiac surgery service would have a risk score based on the sum of the regression coefficients from those two services.

Homogeneous subgroups of hospitals with similar risk scores were then identified and these groupings were used to reclassify the original hospital levels. To demonstrate the impact of re-classification, Bayesian shrinkage plots [16,17] were created using risk-adjusted rates calculated according to the original and re-classified hospital levels. Shrinkage estimators have been used extensively to derive better estimates of the true infection rates in hospitals. They minimize the mean squared error of parameter estimates between hospitals, adjust for variation in sample size and account for regression to the mean for individual hospitals. Statistical analyses were performed using Stata 10.1 software (StataCorp, College Station, TX, USA) and R 2.7.1 (R Core Development Team, Vienna, Austria).

## Results

**Figure 1:**
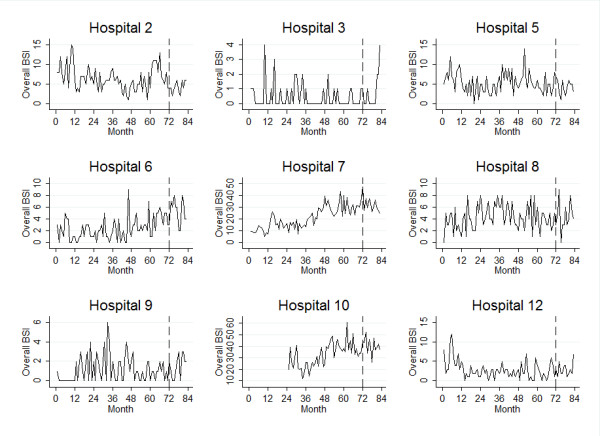
**Longitudinal plots of overall bloodstream infection rates in a sample of public hospitals in Queensland, Australia**. A dashed vertical line splits training and validation subsets of the data, collected from 2001 to 2007. Hospitals 5, 7, 8 and 10 were level I hospitals and the remaining hospitals were level II hospitals.

The mean BSI rate per month by outcome type and mean number of patient days per month, stratified by original hospital levels are displayed in Table 1. The results indicated that level I hospitals had the highest rates of BSI and they were the busiest group of hospitals with the highest number of patient days per month. Level III hospitals tended to have a very low number of infections per month. Across all levels, the outcomes of IVD-STAPH and MRSA were infrequent relative to OBSI and STAPH. Plots of numbers of OBSI per month are presented for selected hospitals in Figure 1.

**Table 1:** Average monthly numbers of bloodstream infections and patient days by hospital level in Queensland, Australia, 2001-2007.

Hospital level by outcome	Mean	Standard Deviation	Range (min to max)
**OBSI**			
**1**	13.7	12.6	0 to 61
**2**	2.6	2.7	0 to 18
**3**	0.7	1.1	0 to 6
**STAPH**			
**1**	2.6	2.4	0 to 13
**2**	0.8	1.1	0 to 8
**3**	0.2	0.4	0 to 3
**IVD-STAPH**			
**1**	0.9	1.2	0 to 8
**2**	0.2	0.5	0 to 3
**3**	0.1	0.2	0 to 1
**MRSA**			
**1**	0.6	0.9	0 to 4
**2**	0.2	0.5	0 to 3
**3**	0.03	0.2	0 to 1
**Patient days**			
**1**	13464.1	6325.8	4775 to 26225
**2**	6564.0	3232.6	2205 to 17929
**3**	2476.7	1026.6	915 to 9635

### Regression models

For the OBSI model (Table 2), the QIC suggested an autoregressive (AR) structure of lag one was most suitable for the correlation structure of the GEE. Four risk services and four protective services were found. The concordance correlation between the observed and fitted values on the validation sample was 0.93 (95% CI .91 to .94), which suggested strong evidence of agreement for the OBSI model. The *c*-index was 0.83 (95% CI .81 to .86) which indicated the model had a high ability to discriminate. The highest OBSI rates among all hospitals were found in hospital 7 and 10 (Figure 1); these two hospitals had all four risk services found by the GEE Poisson model. The risk for OBSI in these hospitals may be compounded as these four risk services were found together.

**Table 2:** Incidence rate ratios of overall blood stream infections for services provided by public hospitals in Queensland, Australia.

Medical services		GEE Poisson with a log link^a^			
		IRR	SE^†^	(95% CI)^†^	*P*-value
**AIDS**		2.14	0.63	(1.20 to 3.82)	.010
**Alcohol/Drugs**		0.52	0.15	(0.29 to 0.93)	.028
**Coronary care**		0.74	0.05	(0.66 to 0.84)	< .001
**Hospice care**		0.84	0.07	(0.71 to 0.99)	.041
**Infectious diseases**		2.72	0.45	(1.97 to 3.76)	< .001
**Plastic surgery**		0.49	0.07	(0.38 to 0.64)	< .001
**Oncology**		1.60	0.17	(1.29 to 1.98)	< .001
**Bone marrow transplants**		1.52	0.22	(1.14 to 2.03)	.004
					
**QIC**	1844.93				

^a^AR 1 correlation

^† ^exchangeable standard errors were scaled by square root of Pearson χ^2^-based dispersion.

For the STAPH models, the parsimonious GEE Poisson model with an autoregressive structure of two lags is shown in Table 3. Three risk services and three protective services were found. The concordance correlation was 0.73 (95% CI .68 to .78). Thus there was moderate level of agreement. The *c*-index was 0.82 (95% CI .78 to .86) indicating a high level of discrimination.

**Table 3:** Incidence rate ratios of *Staphylococcus aureus *bloodstream infections for services provided by public hospitals in Queensland, Australia.

Medical services		GEE Poisson with a log link			
		IRR	SE^†^	(95% CI)^†^	*P*-value
**Alcohol/Drugs**		0.81	0.07	(0.69 to 0.95)	.011
**Diabetes**		0.53	0.11	(0.36 to 0.80)	.002
**Infectious diseases**		3.91	0.82	(2.59 to 5.90)	< .001
**Plastic surgery**		0.57	0.09	(0.42 to 0.79)	.001
**Maintenance renal dialysis**		1.82	0.20	(1.47 to 2.26)	< .001
**Oncology**		1.73	0.14	(1.47 to 2.04)	< .001
					
**QIC**	817.95				

^a^AR 2 correlation

^† ^exchangeable standard errors were scaled by square root of Pearson χ^2^-based dispersion.

IVD-STAPH had very low monthly counts with a large proportion of zeroes (75.9%). The QIC results suggested an autoregressive structure of lag 2 was most suitable. The parsimonious GEE model with AR 2 correlation is shown in Table 4. Three risk services and one protective service were identified. The concordance correlation was 0.58 (95% CI .50 to .65), which indicated a low level of agreement mainly due to the model being unable to predict a substantial number of observed zeroes. However, the *c*-index was moderately high at 0.78 (95% CI .72 to .85) which suggested sufficient ability to discriminate between lower and higher infection rates among hospitals.

**Table 4:** Incidence rate ratios of intravascular device related *Staphylococcus aureus *blood stream infections for services provided by public hospitals in Queensland, Australia.

Medical services		GEE Poisson with a log link^a^			
		IRR	SE^†^	(95% CI)^†^	*P*-value
**Infectious diseases**		3.35	0.89	(1.99 to 5.64)	< .001
**Plastic surgery**		0.46	0.13	(0.26 to 0.81)	.008
**Maintenance renal dialysis**		1.50	0.25	(1.08 to 2.08)	.016
**Oncology**		1.89	0.26	(1.44 to 2.48)	< .001
					
**QIC**	507.79				

^a^AR 2 correlation

^† ^exchangeable standard errors were scaled by square root of Pearson χ^2^-based dispersion.

MRSA had very low monthly counts with a large proportion of zeroes (79.7%) and a maximum monthly count of four events. The QIC suggested an independence structure adequately reflected the correlation structure. The parsimonious logistic regression model is shown in Table 5. Three risk services and one protective service were found. The Hosmer-Lemeshow goodness of fit test with 10 groups suggested the model fitted adequately (χ^2^(8) = 6.74, *P *= .565). The AUC suggested good discrimination between observed and fitted values (AUC = .81, exact 95% CI .79 to .83).

**Table 5:** Odds ratios of methicillin-resistant *Staphylococcus aureus *bloodstream infections for services provided by public hospitals in Queensland, Australia.

Medical services		GLM binomial with a logit link			
		OR	SE	(95% CI)	*P*-value
**Acute renal dialysis**		2.77	0.58	(1.83 to 4.18)	< .001
**Cardiac surgery**		1.59	0.31	(1.08 to 2.32)	.017
**Infectious diseases**		3.12	1.04	(1.62 to 5.99)	.001
**Plastic surgery**		0.48	0.16	(0.25 to 0.93)	.029
					
**AIC**	867.04				
**QIC**	258.56				

### Level re-classification based on risk scoring

Direct hospital-specific risk adjustment using the IVD-STAPH model was not recommended due to the low concordance correlation. Note, a GEE negative binomial model and a zero-inflated Poisson (ZIP) model [18] were also fitted for IVD-STAPH but resulted in similarly low concordance correlations. Therefore, the risk scoring approach was used for IVD-STAPH, and MRSA.

**Figure 2:**
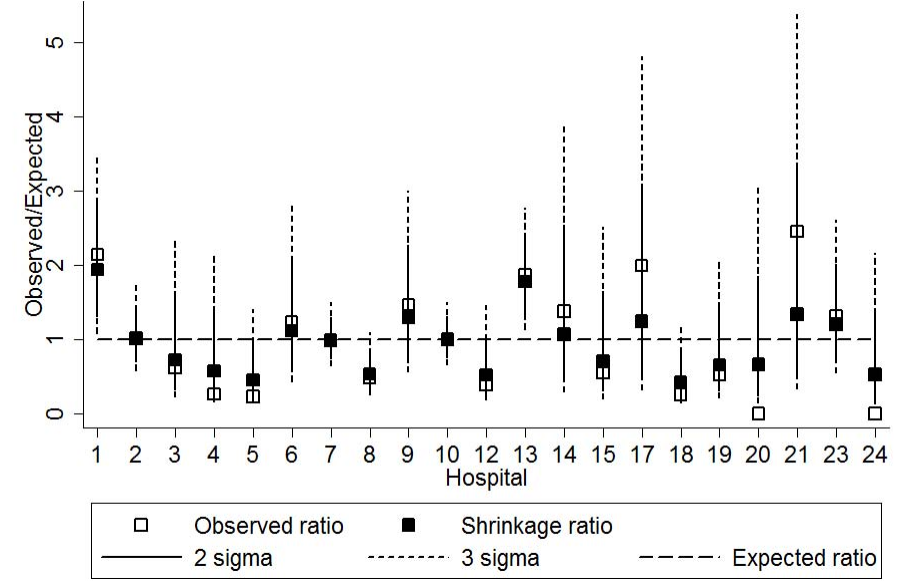
**Bayesian shrinkage Observed/Expected plot of methicillin-resistant *Staphylococcus aureus *bloodstream infection rates in public hospitals in Queensland, Australia, 2003-2007, with risk adjustment by crude hospital levels**.

Table 6 demonstrates the calculation of the risk score, and subsequent hospital reclassification for MRSA. Figure 2 shows a Bayesian shrinkage plot for five years of MRSA surveillance data with risk adjustment by the original hospital levels. The two and three standard deviation boundaries (sigma control limits) can be used to identify which hospitals have significantly over or under-performed relative to their peers. Hospitals 1 and 12 performed significantly worse than average at the 2 and 3 sigma control limits.

**Table 6:** Reclassification of hospital levels for methicillin-resistant *Staphylococcus aureus *bloodstream infection risk-adjustment in public hospitals in Queensland, Australia

Hospital ID	MRSA risk score	Reclassified level	Original level	Acute Renal Dialysis (Coeff 1.02)	Cardiac Surgery (Coeff .46)	Infectious Diseases (Coeff 1.14)	Plastic Surgery (Coeff -.73)
**1**	2.2	1	2	Yes	No	Yes	No
**13**	1.9	1	1	Yes	Yes	Yes	Yes
**7**	1.9	1	1	Yes	Yes	Yes	Yes
**10**	1.4	1	1	Yes	No	Yes	Yes
**2**	1.4	1	2	Yes	No	Yes	Yes
**12**	1.0	2	2	Yes	No	No	No
**9**	1.0	2	2	Yes	No	No	No
**14**	1.0	2	3	Yes	No	No	No
**17**	1.0	2	3	Yes	No	No	No
**8**	0.5	2	1	No	Yes	No	No
**5**	0.4	2	1	No	No	Yes	Yes
**6**	0.3	2	2	Yes	No	No	Yes
**19**	0	3	2	No	No	No	No
**4**	0	3	1	No	No	No	No
**15**	0	3	3	No	No	No	No
**16**	0	3	3	No	No	No	No
**20**	0	3	3	No	No	No	No
**21**	0	3	3	No	No	No	No
**23**	0	3	2	No	No	No	No
**18**	0	3	2	No	No	No	No
**24**	0	3	3	No	No	No	No

Yes = Medical service available

No = Medical service not available

Reclassification is based on risk scores derived from the medical services provided and coefficients from logistic regression models of the relationship between each medical service and the monthly occurrence of infection in each hospital.

**Figure 3:**
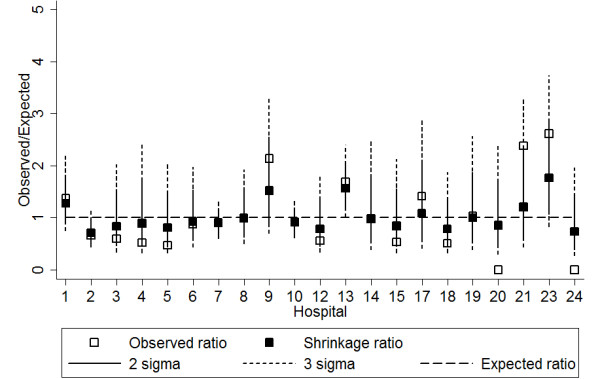
**Bayesian shrinkage Observed/Expected plot of methicillin-resistant *Staphylococcus aureus *bloodstream infection rates in public hospitals in Queensland, Australia, 2003-2007, with risk adjustment by reclassified levels**. Reclassification is based on a risk score derived from the services provided by each hospital.

Figure 3 shows that when risk adjustment was performed using the re-classified levels (based on the risk score), hospital 12 remained an outlier but hospital 1 was clearly within the 2 and 3 sigma control limits. Thus hospital 1, which had been re-classified from level II to level I (Table 6), was under control. When deriving the shrinkage ratios for MRSA, the estimate of the between-hospital variation of the true rates was obtained. With the original levels, the variation between hospitals was 0.332. With the new levels, the variation decreased to 0.138.

## Discussion

This study aimed to develop and evaluate risk adjustment for BSI rates at a hospital level, based on services provided by those hospitals. Risk adjustment is clearly necessary given the large differences in rates between hospital levels across all four infection outcomes (Table 1; Figure 1). Our results suggest that hospital-specific risk adjustment based on medical services provided is strongly recommended for OBSI and STAPH. Expected infection counts (calculated using patient day denominators), may be obtained directly from the risk-adjustment models. By contrast, hospital-level risk adjustment with a risk score approach is recommended for IVD-STAPH and MRSA in lieu of direct hospital-specific risk adjustment. These methods may be used to derive less biased observed-expected ratios of monthly BSI than the crude approach currently being used, where risk adjustment is based on the hospital level, and CHRISP is currently implementing risk adjustment using the models presented in this report.

The risk-adjusted ratios may be implemented on the *y*-axis in funnel plots [19] and Bayesian shrinkage plots for continuous quality improvement. Shrinkage plots for MRSA demonstrate that hospital 1, reported as an outlier using the original hospital level classification, was found to be under control using our re-classified hospital levels. Table 6 indicated that the hospital, originally classified as a level 2 hospital, was reclassified as a level 1 hospital using the risk score based on the logistic regression model. This was because the hospital offered acute renal dialysis and infectious diseases services, which were the highest risk services found in the MRSA model. Hospital 1 actually had the highest risk score among all hospitals. It was also found that between-hospital variation in rate estimates was higher for the original, crudely adjusted values than in the new risk-adjusted values. This is further evidence to support the reclassification, as the new levels produced a more homogenous group of true rates within each level, and demonstrates that the risk scoring approach has had a significant impact on the interpretation of observed MRSA rates.

AIDS, infectious diseases, oncology, renal dialysis, cardiac surgery and transplant services were found to be high risk services for BSI in one or more models. This is unsurprising given the compromised immune state of most patients cared for by AIDS, oncology and transplant services, and the large number of invasive procedures conducted in oncology, renal dialysis and cardiac surgery wards. Infectious disease services were highly collinear with intensive care services and the finding of infectious disease services as high risk could relate to the health status and number of invasive procedures experienced by patients in intensive care units and other collinear services. Another possibility is that hospitals with infectious disease units might perform better at BSI surveillance, with a higher probability of identifying and reporting BSI cases. This requires further investigation. We note that exclusion of the three level II hospitals with missing data potentially reduced the power of the statistical models to identify risk and protective services, and might have introduced an immeasurable source of bias.

Although hospital-level risk adjustment based on hospital services is a more objective and refined approach than that based on crude hospital levels, hospital services remain an indirect indicator of patient case-mix. Use of service-specific infection rates (which were not available in the current study) or attribute data of individual patients (also not available for the general patient population) would facilitate a more accurate and robust approach to risk adjustment. Further research will focus on developing risk-adjustment models that incorporate more sophisticated denominators such as central line-days to calculate BSI rates [20]. CHRISP is in the process of initiating a pilot of central line-day data collection within a major hospital. It is possible that variation in surveillance quality could contribute to observed variation in BSI rates between hospitals. We cannot capture this in our models but if a hospital signals (i.e. has a higher than expected rate), an investigation should be conducted and this will determine if the signal is a reporting artifact or a result of an infection control break down. While our validation results suggest that the models were robust over time, we do not have data available from another geographical area (e.g. Australian state) for external validation purposes but that is something we wish to investigate in the future.

## Conclusion

The results of the models are generalizable to the network of public hospitals in Queensland. While the estimates of the models themselves may not be generalizable to other healthcare systems with different patient case mixes and organization of medical services, the statistical methods of risk-adjustment presented here are widely applicable to other healthcare systems that collect BSI surveillance data at an aggregated hospital level. The Australian government is currently mandating *S. aureus *BSI as a key performance indicator, and risk-adjustment will be essential to ensure that hospitals that offer high risk services will not be unfairly penalized given their underlying propensity of their patients to develop BSI. Therefore, the imperative is great for more objective methods of risk adjustment such as the approach outlined in this report.

## Competing interests

The authors declare that they have no competing interests.

## Authors' contributions

ACAC and ENCT designed the study. ENCT, MAH and MAJ conducted the statistical analysis. APM MW and ACAC advised on the clinical aspects of the study. ENCT and ACAC wrote the manuscript with major contributions from all co-authors. All authors have read and approved the final manuscript.

## Pre-publication history

The pre-publication history for this paper can be accessed here:

http://www.biomedcentral.com/1471-2334/9/145/prepub
